# Racial Differences in Strength of Associations Between Colorectal Cancer Screening, Area Deprivation, Demographics, and Clinical Characteristics

**DOI:** 10.31486/toj.23.0012

**Published:** 2023

**Authors:** Eboni G. Price-Haywood, Jeffrey H. Burton

**Affiliations:** ^1^ Ochsner-Xavier Institute for Health Equity and Research, New Orleans, LA; ^2^ Center for Outcomes Research, Ochsner Clinic Foundation, New Orleans, LA; ^3^ The University of Queensland Medical School, Ochsner Clinical School, New Orleans, LA

**Keywords:** *Colorectal cancer screening*, *early detection of cancer*, *healthcare disparities*, *social determinants of health*

## Abstract

**Background:** In Louisiana, colorectal cancer (CRC) incidence and mortality exceed national rates. Census tract, sex, and racial disparities across the state are well documented. This study examined whether there were subpopulation differences in associations between CRC screening, area deprivation index (ADI), and patient characteristics.

**Methods:** This retrospective observational study included patients aged 50 to 75 years who received care within Ochsner Health in Louisiana between July 1, 2012, and December 31, 2020. Logistic regression models were used to generate adjusted odds ratios (95% CI).

**Results:** A total of 75,344 patients met eligibility criteria for inclusion in the data analysis (60% female, 36% Black, 56% with spouse/partner, 42% Medicare/Medicaid,17% living in high deprivation areas, 41% with 2+ chronic conditions, 56% never smoked, 51% obese). Living in areas with less deprivation (state decile 1-3 vs 8-10: 1.19 [1.14-1.24]), number of comorbidities (3+ conditions: 1.15 [1.12-1.17]), and prior outpatient visits (1.63 [1.58-1.67]) increased odds of CRC screening. Male sex (0.82 [0.79-0.84]), age group 55 to 59 years (0.97 [0.95-0.99]), and Medicaid insurance (0.89 [0.86-0.92]) decreased odds of screening. ADI was collinear with sex, race, marital status, body mass index, and smoking status. In subgroup analyses, between-group differences in strength of associations of CRC screening with ADI and patient characteristics varied most prominently by race.

**Conclusion:** There may be an unmeasured social context explaining persistent racial differences among factors associated with CRC screening. A combination of census tract and individual-level social determinants may guide population health management for at-risk subpopulations.

## INTRODUCTION

Between 1970 and 2017, overall colorectal cancer (CRC) incidence and mortality rates declined in the United States.^1^ Nonetheless, notable cancer disparities across demographic groups, which have largely been attributed to socioeconomic inequalities, result in differences in access to early detection and timely treatment. Geographic regions of the South, Midwest, and Appalachia have among the highest incidence and mortality rates of CRC. While older age groups account for the majority of CRC cases, the rates among lower age groups have increased. Males compared to females and non-Hispanic Blacks compared to other racial/ethnic groups have higher rates of CRC.^1^

A complex array of factors (patient, provider, health system, community, public policy) impact cancer screening utilization. CRC screening is lowest among the 50 to 54 years age group, individuals with less than a high school education, low-income households, the uninsured, and recent immigrants.^1-4^ Having usual sources of care, provider recommendation, adequate insurance coverage, convenience of accessing services, and related logistical factors (eg, transportation, scheduling) are associated with cancer screening adherence.^1,5-9^ Additionally, marital status,^5,10,11^ being underweight or obese,^12-14^ tobacco use,^15,16^ and number of comorbidities^8,17^ are associated with screening utilization.

Many of the aforementioned individual characteristics highlight the impact of social determinants of health (SDoH) on the uptake of CRC screening. As health systems shift toward value-based care and population health management, interventions aimed at improving CRC screening must address SDoH barriers. To do so effectively will require optimization of SDoH data collection while delivering care—a process that is not yet routine in health care. An alternative option is to use area- or neighborhood-level measures such as the area deprivation index (ADI) or social vulnerability index (SVI) to help identify at-risk populations.^18,19^ The ADI is a measure of the socioeconomic disadvantage of a geographic area based on rates of poverty, housing characteristics, employment, and education.^18^ The SVI, traditionally used for planning responses to public health crises, assesses socioeconomic status, household composition and disability, minority status and language, and housing and transportation.^19^ Previous studies have demonstrated that living in areas with higher deprivation and/or social vulnerability is associated with lower preventive health behaviors such as cancer screening rates.^20-22^ Therefore, geographic measures of SDoH may be useful adjuncts for identifying populations at risk for CRC screening nonadherence.

In the state of Louisiana, where the CRC incidence and mortality rates (45.1 and 15.6 per 100,000 per year, respectively) exceed the national rates,^23^ 47 census tracts across 26 (of 64 total) parishes have a statistically higher incidence of CRC compared to the rest of the state.^24^ Notably, Black males have the highest CRC incidence and mortality rates in Louisiana.^25^ In general, the Louisiana population has sex-related disparities in cancers (including CRC) associated with modifiable risk factors of tobacco use (males > females) and obesity (females > males).

These disparities in Louisiana persist despite the overall declining CRC incidence and mortality rates for the general population which has been attributed to increased CRC screening. To close the gap, health systems may need to tailor population health management to specific subpopulations. The objectives of this study were to examine the following in the Ochsner Health patient population: (1) whether the relationship between CRC screening adherence, ADI, and patient characteristics differs among subpopulations by sex, race, marital status, body mass index (BMI), and smoking status; and (2) whether the direction and strength of the associations differ among subpopulations by sex, race, marital status, BMI, and smoking status.

## METHODS

### Study Design, Setting, and Population

This study is a retrospective cohort analysis of medical records for patients aged 50 to 75 years who received care within Ochsner Health in Louisiana, between July 1, 2012, and December 31, 2020. Ochsner operates 47 hospitals and 370 health and urgent care centers across Louisiana, Mississippi, Alabama, and the Gulf South area. The index observational period was July 1, 2013, to December 31, 2019. This time interval allowed for 1-year pre-index and post-index periods for all patients within the larger study period. The pre-index period ended in 2019 given the onset of the coronavirus disease 2019 pandemic in 2020 and the subsequent decrease in availability of outpatient procedures. Patients who met the following criteria were included in the analysis: (1) within the age range of 50 to 74 years as specified in the 2016 US Preventive Services Task Force (USPSTF) CRC screening guidelines;^26^ (2) no diagnosis of CRC at the beginning of their respective index period; (3) residential address in Louisiana; (4) 3 or more primary care encounters during the follow-up period (ie, an established patient with continuity of primary care). Notably, the 2018 USPSTF guidelines that expanded the age range to 45 to 74 years had not yet been implemented at Ochsner Health at the time of the study. Insurance coverage for CRC screening in the 45 to 49 years age group was also not universal. This study was approved by the Ochsner Health Institutional Review Board.

### Health System Population Health Management

In accordance with the Community Preventive Services Task Force recommendations,^27^ Ochsner Health employs a multicomponent intervention to increase CRC screening (provider reminders and performance feedback, reminders for average-risk, age-eligible patients). The health system uses the health maintenance registry tool in the Epic (Epic Systems Corporation) electronic health record (EHR) system to prompt primary care physicians at the point of care to review patients’ status of adherence to clinical guidelines. CRC screening registries were activated in 2015 in accordance with the USPSTF guidelines. Providers also have patient panel performance dashboards in Epic. In 2016, nurse care coordinators were added to the care teams to contact patients with reminders and reconcile screening status. Screenings completed within the health system automatically update the registry. For screenings completed elsewhere, the health system uses external insurance claims and Epic's Care Everywhere function to verify completion of health care maintenance. If the code documented in Care Everywhere is mapped to a procedure in the Ochsner Health Epic system, the health care maintenance registry will indicate that the procedure was completed. If the procedure was done outside of Ochsner and the procedure code is not captured by Care Everywhere, nurse care coordinators retrieve information from the outside organization, create an external result, and scan the procedure note to the order.

### Main Outcome–Adherence to Cancer Screening

CRC screening guideline adherence was defined as having completed one of the following: (1) colonoscopy every 10 years, (2) fecal immunochemical test (FIT) every year, (3) stool DNA test with FIT component every 1 to 3 years, (4) high-sensitivity guaiac fecal occult blood test (gFOBT) every year, (5) flexible sigmoidoscopy every 5 years + high-sensitivity gFOBT every 3 years. Historic patient records were extracted from the health system's enterprise data warehouse. For each patient, the index date was the date of the first primary care encounter at which the patient qualified for screening. The end of follow-up was defined as the date at which each patient aged out of the recommended age range, the date of cancer diagnosis, or the end of the study.

Based on the recommended time intervals for procedures in the cancer screening guidelines, procedures documented in patient records were used to determine the date when a patient became adherent to guidelines and the date when adherence lapsed. Adherence was assessed on an annual basis. A patient was adherent for a given calendar year if the patient met screening criteria on any given day within a calendar year. A patient was not adherent for a given calendar year if the patient did not meet screening criteria in that year or the patient met the criteria for only a portion of the year. The assessment for annual adherence was only for full calendar years during the patient follow-up period. The assessment period started with first full calendar year following the index visit and ended with last full calendar year prior to the end of follow-up. The study did not assess years in which the patient did not qualify for screening for the duration of the year.

### Covariates of Interest

Patients were assigned an ADI 2019 national percentile ranking based on their home residence. The ADI metrics were available for census block groups with national percentile ranking (0=least disadvantaged/deprived, 100=most disadvantaged/deprived). The following variables were extracted from the EHR: age, self-reported sex and race/ethnicity, marital status, insurance type, number of comorbidities, last recorded BMI, smoking status, outpatient encounters in the pre-index year, and years in which patient had encounters in the health system.

### Statistical Analysis

All continuous measures included as independent variables are categorized with consideration for meaningful interpretations. Records with missing data for any independent variable are excluded from analysis. At the time of this study, only a portion of Ochsner patients had their documented residential address geocoded for assignment of ADI metrics corresponding to a given block group. Characteristics of patients both with and without geocoded addresses were examined to ensure minimal bias was introduced when assessing associations with ADI. Mixed-effects logistic regression models were used to generate unadjusted and adjusted odds ratios (ORs) and 95% CIs. ORs represent the associations between independent variables and adherence to screening guidelines across the follow-up period only for patients with ADI geocoding available at the time of this study. The multivariate response is composed of a series of patient-specific vectors of binary indicators of screening adherence at each calendar year in the study period. All models include a categorical fixed effect for year and a patient-level random intercept term. At the patient level, a compound symmetric structure is imposed on the covariance matrix to model within-subject correlation over time. Unadjusted analyses consist of incorporating a single fixed effect corresponding to the variable under investigation. For adjusted analyses, multivariable models incorporate a fixed effect for ADI and fixed effect covariates for selected demographic and clinical variables. For variables with more than 2 levels, adjustments via simulation for multiple comparisons are used to control inflation of type I error rate. Slight to moderate collinear associations were observed between ADI with race/ethnicity, BMI, smoking status, insurance, and Charlson Comorbidity Index. Therefore, subgroup analyses were conducted for select variables (sex, race/ethnicity, marital status, BMI, smoking status). This approach avoids fluctuations in the precision of coefficient estimates of correlated variables which may be observed when included in the same model. *P* values are from tests of interactions with the stratifying variables. All analyses were conducted using SAS v. 9.4 (SAS Institute Inc).

## RESULTS

### Patient Characteristics

Nearly 200,000 patients were eligible for CRC screening during the study period (Table 1). A total of 75,344 patients had ADI geocoding recorded and met eligibility criteria for inclusion in the data analysis. No statistically significant demographic or clinically significant differences were observed between patients with vs without ADI geocoding. In the group with ADI geocoding available, 59.8% of patients were female, more than one-third self-identified as Black, and only 2% were Hispanic. The proportions of age groups were similar across strata. In the group with ADI geocoding available, approximately 56% were married or had a partner, 42% were publicly insured, and 17% had residences in areas of high deprivation. The mean Charlson Comorbidity Index average was 2.3 for the ADI group; 41% of the patient cohort had 2 or more chronic conditions; 56% never smoked tobacco; and 51% were obese. Less than 50% had a pre-index outpatient encounter within the health system.

**Table 1.** Characteristics at Index Visit of Patients Eligible for Colorectal Cancer Screening

	Area Deprivation Index Geocoding Available	
Variable	Yes, n=75,344	No, n=122,633
Age, years		
50-54	22,051 (29.3)	35,872 (29.3)
55-59	16,120 (21.4)	26,213 (21.4)
60-64	15,529 (20.6)	24,015 (19.6)
65+	21,644 (28.7)	36,533 (29.8)
Sex		
Female	45,072 (59.8)	69,543 (56.7)
Male	30,270 (40.2)	53,090 (43.3)
Race^a^		
White	46,141 (61.2)	89,434 (72.9)
Black	27,180 (36.1)	29,428 (24.0)
Ethnicity^a^		
Hispanic	1,834 (2.4)	2,562 (2.1)
non-Hispanic	73,042 (96.9)	119,272 (97.3)
Marital status^a^		
Married/partnered	42,030 (55.8)	77,551 (63.2)
Divorced/separated/widowed	14,841 (19.7)	19,455 (15.9)
Single	18,265 (24.2)	25,120 (20.5)
Insurance		
Commercial	40,212 (53.4)	70,500 (57.5)
Medicare	26,615 (35.3)	41,137 (33.5)
Medicaid	4,735 (6.3)	4,819 (3.9)
Uninsured/self-pay	3,002 (4.0)	4,890 (4.0)
Other	780 (1.0)	1,287 (1.0)
Area deprivation index state decile ranking		
1-3 (least deprived)	30,869 (41.0)	–
4-7	31,597 (41.9)	–
8-10 (most deprived)	12,878 (17.1)	–
Charlson Comorbidity Index, mean (SD)	2.3 (2.8)	2.1 (2.8)
Number of comorbidities^a^^,^^b^		
0	26,214 (34.8)	50,000 (40.8)
1	18,207 (24.2)	29,335 (23.9)
2	12,616 (16.7)	18,799 (15.3)
3+	18,306 (24.3)	24,499 (20.0)
Smoking status^a^		
Current	12,594 (16.7)	19,097 (15.6)
Former	19,963 (26.5)	31,598 (25.8)
Never	42,546 (56.5)	71,429 (58.2)
Body mass index^a^		
Underweight	648 (0.9)	1,168 (1.0)
Normal	13,042 (17.3)	22,955 (18.7)
Overweight	23,151 (30.7)	39,687 (32.4)
Obese	38,038 (50.5)	57,990 (47.3)
Outpatient encounter in pre-index year	35,793 (47.5)	53,330 (43.5)

^a^Categories corresponding to other/unknown are not displayed.

^b^Comorbidities: cancer, cerebrovascular disease, cardiovascular disease, chronic pulmonary disease, liver disease, renal disease, HIV, diabetes, dementia, rheumatic disease, hemiplegia/paraplegia.

Note: Data are presented as n (%) unless otherwise indicated.

### Collinearity of Area Deprivation Index With Other Characteristics

A small number of variables considered for inclusion as covariates in the multivariable model to assess the association between ADI national percentile ranking and screening adherence showed slight to moderate bivariate associations with ADI. The strongest relationship observed was between race/ethnicity and ADI (Cramér V=0.33). Other nominal variables associated with ADI were marital status (V=0.12), smoking status (V=0.09), and sex (V=0.04). Additionally, BMI showed a slight linear association with ADI (Pearson *ρ*=0.14). Although the associations are mild, subgroup analyses were carried out to investigate the relationship between ADI and cancer screening within levels of each of these variables rather than including them as covariates in the full sample multivariable model.

### Characteristics Associated With Screening Adherence

In this study population, living in less-deprived areas (lower ADI), having a higher number of comorbidities, and having prior outpatient visits were associated with higher odds of CRC screening, while age group 55 to 59 years, male sex, and Medicaid were associated with lower odds in the adjusted analysis (Table 2). The strongest predictor of screening was prior outpatient encounters.

**Table 2.** Unadjusted and Covariate-Adjusted Odds Ratios of Colorectal Cancer Screening Adherence

Variable	Unadjusted Odds Ratio (95% CI)	Covariate-Adjusted Odds Ratio (95% CI)
Area deprivation index state decile ranking vs most deprived (8-10)		
1-3	1.22 (1.17-1.28)^a^	1.19 (1.14-1.24)^a^
4-7	1.24 (1.18-1.29)^a^	1.20 (1.14-1.25)^a^
Age group vs 50-54 years		
55-59	0.97 (0.95-0.98)^a^	0.97 (0.95-0.99)^a^
60-64	0.98 (0.96-1.00)	0.97 (0.95-1.00)
65+	1.02 (0.99-1.05)	0.98 (0.95-1.02)
Sex, male vs female	0.81 (0.78-0.83)^a^	0.82 (0.79-0.84)^a^
Insurance type vs commercial		
Medicaid	0.88 (0.85-0.91)^a^	0.89 (0.86-0.92)^a^
Medicare	1.04 (1.02-1.06)^a^	1.02 (1.00-1.04)
Other	0.94 (0.88-1.01)	0.95 (0.89-1.01)
Uninsured/self-pay	0.95 (0.92-0.99)^a^	0.97 (0.93-1.01)
Number of comorbidities vs 0		
1	1.06 (1.04-1.08)^a^	1.05 (1.03-1.07)^a^
2	1.14 (1.11-1.16)^a^	1.12 (1.10-1.15)^a^
3+	1.17 (1.15-1.20)^a^	1.15 (1.12-1.17)^a^
Preindex outpatient encounter, yes vs no	1.69 (1.64-1.74)^a^	1.63 (1.58-1.67)^a^

^a^Statistically significant whereby the 95% CI does not contain 1.0. The adjusted model contains area deprivation index, age, sex, insurance, number of comorbidities, pre-index outpatient encounter.

#### Sex

**Figure 1.**
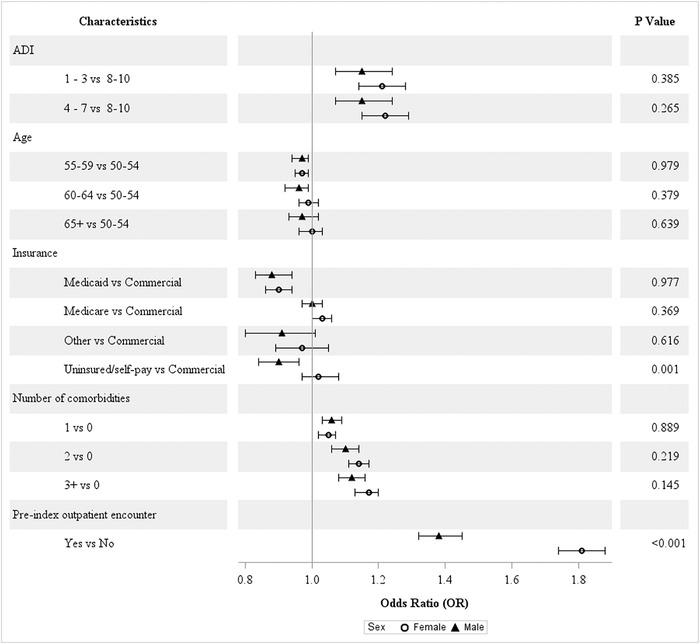
**Covariate-adjusted odds ratios of colorectal cancer screening adherence by sex.** ADI, area deprivation index.

Among females compared to males, significant differences in the odds of CRC screening were only observed for uninsured status and prior outpatient encounters (Figure 1). Lack of insurance significantly decreased the odds of male patient adherence to screening. Prior outpatient encounters significantly increased the odds of screening for both groups; however, the strength of the association was greater for female patients.

#### Race

**Figure 2.**
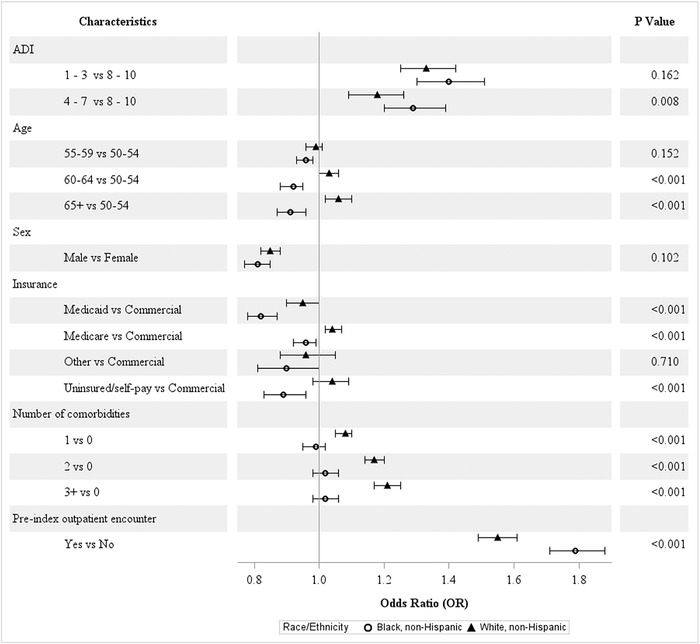
**Covariate-adjusted odds ratios of colorectal cancer screening adherence by race/ethnicity.** ADI, area deprivation index.

Among Whites compared to Blacks, significant group differences were observed for all characteristics except sex (Figure 2). The direction of the associations with ADI and prior outpatient encounters was similar; however, the strength of the associations differed, with Blacks having relatively higher odds of adherence compared to Whites. Older age increased the odds of screening among Whites and decreased the odds among Blacks. Within each insurance type, Whites had relatively higher odds of CRC screening. Notwithstanding, Medicaid insurance decreased the odds of adherence among both groups. Whites also had relatively higher odds of screening with increasing number of comorbidities.

#### Marital Status, BMI, and Smoking Status

**Figure 3.**
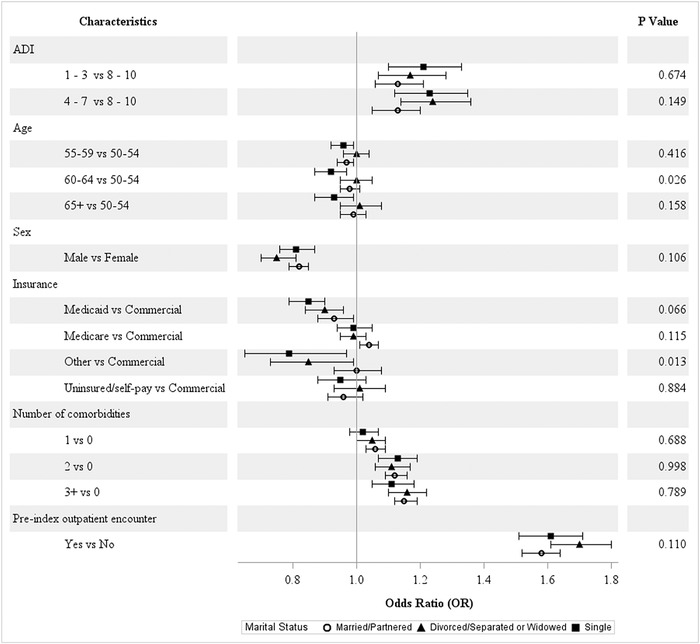
**Covariate-adjusted odds ratios of colorectal cancer screening adherence by marital status.** ADI, area deprivation index.

**Figure 4.**
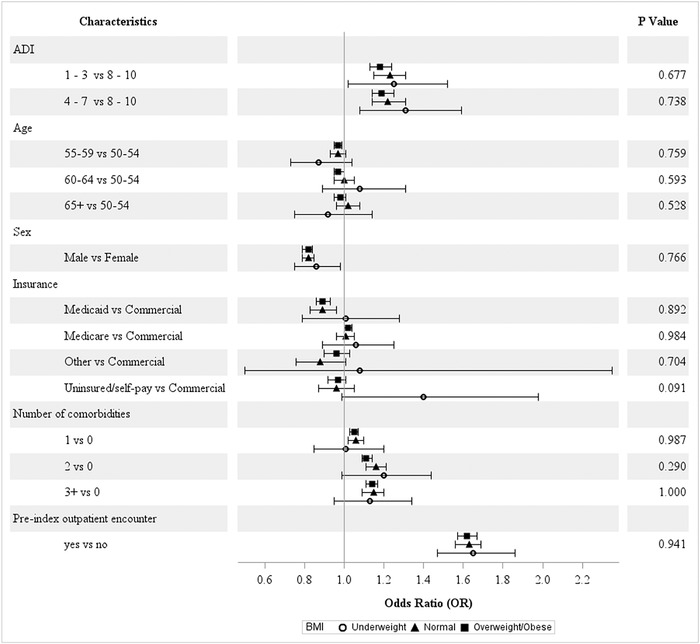
**Covariate-adjusted odds ratios of colorectal cancer screening adherence by body mass index (BMI).** ADI, area deprivation index.

**Figure 5.**
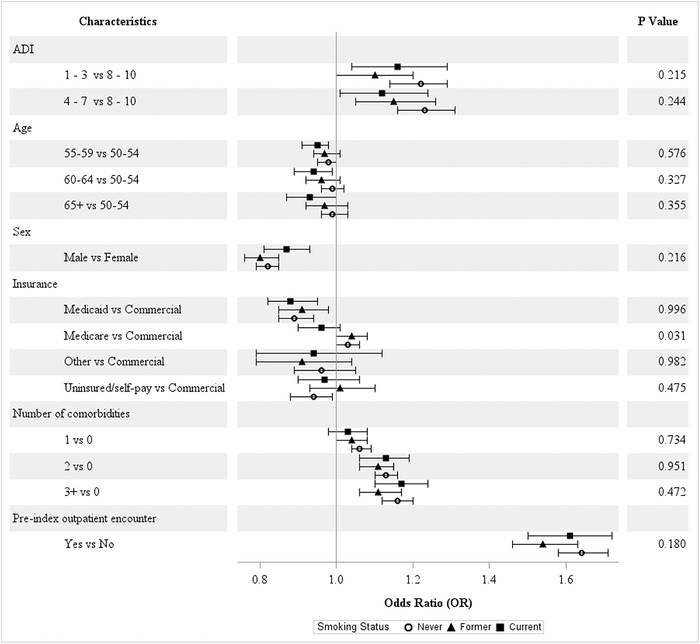
**Covariate-adjusted odds ratios of colorectal cancer screening adherence by smoking status.** ADI, area deprivation index.

Across the strata for marital status (Figure 3), age and other insurance types (eg, workers’ compensation) significantly reduced the odds of screening adherence among single patients. No differences in patterns were observed for the other characteristics. No significant differences were observed for BMI categories (Figure 4). For the smoking status categories, a significant difference was observed only for Medicare vs commercial insurance (Figure 5).

## DISCUSSION

In this study of a single health system in Louisiana, living in areas with less deprivation, having an increased number of comorbidities, and having prior outpatient visits increased the odds of CRC screening. In contrast, male sex, age group 55 to 59 years, and Medicaid insurance decreased the odds of CRC screening. The strongest predictor of screening was prior outpatient encounters. Collinearity of ADI with sex, race/ethnicity, marital status, BMI, and smoking status highlights the social context of risk factors for CRC and predictors of CRC screening utilization. Subgroup analyses demonstrated that between-group differences in the strength of the relationships between CRC screening adherence, ADI, and patient characteristics varied most prominently by race.

This study confirms previous studies which indicate that where an individual resides influences preventive health behaviors including CRC screening utilization.^20-22^ Although Ochsner Health employs a multicomponent provider-patient intervention to increase CRC screening, this study substantiates the need to incorporate SDoH into population health management strategies. Measures of neighborhood-level deprivation such as the ADI capture conditions that commonly affect people who live in the same area. Even so, there may still be an additional unmeasured social context that explains the relative differential influence of race, even among people who live in similar areas. Individual-level measures of SDoH (eg, financial strain, food insecurity, transportation needs, social connections, stress, physical activity) may be informative. Notably, in 2016, the Centers for Medicare and Medicaid Services (CMS) released Z codes to facilitate documentation of SDoH captured either via self-reported questionnaires or information solicited while delivering care but frequently buried in clinical notes as unstructured data.^28^ Standard coding would help quickly identify target populations similarly to how *International Classification of Diseases, Tenth Revision* codes are used to identify clinical conditions. However, the Z code adoption rate has been slow. A major limitation of Z codes has been lack of specificity in the coding. To mitigate this problem, CMS added new codes for use beginning in fiscal year 2023. Specifically, CMS expanded Z59.8–Other problems related to housing and economic circumstances to capture transportation insecurity (Z59.82), financial insecurity (Z59.86), and material hardship (Z59.87). Organizations such as the American Hospital Association have begun providing guidance to hospital systems to increase the use of Z codes.^29^ Much work needs to be done to improve both the specificity and efficiency with which these codes are adopted into clinical documentation. Effective implementation would facilitate the ability of health systems to generate patient registries that could be used to target population health programming.

Health systems must be cognizant of the strength and limitations of patient- vs neighborhood-level SDoH data when designing patient interventions.^30^ Collecting data directly from patients is likely to be sensitive and specific to their needs; however, sampling bias and social desirability may affect patient responses. For example, Ochsner Health initiated SDoH data collection via a questionnaire embedded in the Epic EHR. At the time of this study, the questions were administered once a year in advance of an upcoming appointment via the patient portal. Less than 10% of the study population completed the questionnaire, and these individuals had a low rate of self-reported social needs. The health system has ramped up its strategy for collecting this information at the point of care using community health workers and case managers in clinics located in medically underserved areas. Using neighborhood-level data increases health system engagement with upstream factors and focuses efforts on subpopulations that could enhance the value of interventions. However, census-level measures lack timely data given the nature of the source. Additionally, there is a risk of reinforcing inequities by inadvertently stigmatizing subpopulations with labels or intervening on behalf of healthier, low-risk individuals in the same neighborhood. Even so, none of the aforementioned limitations should discourage use of SDoH measures. Incorporating SDoH into population health management still has important value.

This study raises questions about the relationship between Medicaid insurance and access to CRC screening. These questions are especially concerning given the relatively small number of patients with Medicaid as their primary insurance in the study population. In 2016, Louisiana expanded Medicaid coverage.^31^ The hypothesis was that the mandated health insurance coverage for preventive services (including cancer screening) would increase access. However, several studies have demonstrated that Medicaid-insured patients have been less likely to complete CRC screening irrespective of Medicaid expansion.^5,32-35^ Bonafede et al showed that among females with at least one health care encounter, patients insured by Medicaid were less likely to complete CRC screening compared to commercially insured patients.^35^ Mojica et al suggested that proximity to endoscopy facilities may be a factor.^33^ Additionally, state-specific policies on reimbursement for office visits may impact CRC screening among the Medicaid-insured population.^36^ As CMS continues to update the Medicaid and CHIP [Children's Health Insurance Program] Managed Care Final Rule (CMS-2390-F),^37^ value-based models for health care delivery reform are likely to emerge. The Louisiana Department of Health has created a Medicaid Managed Care Quality Dashboard to promote health plan transparency and accountability for CRC screening.^38^ A combination of policy, practice, and patient-level interventions may be needed to promote CRC screening in the Medicaid-insured population.

This study has several limitations. The study data were limited to a single health system, limiting external generalizability. Nonetheless, our study findings substantiate the urgent need to focus on continuity of care and on mitigating the impact of SDoH on CRC screening. The data analysis was based on EHR data only. Without insurance claims data, we can only account for events that occurred within the health system. Therefore, annual screening rates may be an underestimate. Common challenges with real-world data sources include missing or mistimed data, suitable capture of endpoints, and need for linkage to other data sources. Although the health system EHR includes an SDoH questionnaire for patient self-reporting, these data elements are not routinely captured for every patient. The ADI was therefore used as proxy. The geocoding was limited to the ADI national percentile (or state decile levels). Therefore, this study could not decipher which components of deprivation (poverty, housing characteristics, employment, or education) have the strongest association with CRC screening behaviors. Only a portion of patients have had their documented residential address geocoded for ADI assessments. Notwithstanding, characteristics of patients both with and without geocoded locations were examined to ensure minimal bias was introduced when assessing associations with ADI.

## CONCLUSION

The social context that drives disparities in CRC incidence and mortality also increases risk for CRC screening nonadherence. More work is needed to efficiently capture patients’ individual-level SDoH to guide tailoring of population health management strategies to at-risk subpopulations. Improving data collection is especially imperative given demonstrable racial differences in health outcomes even among people living in areas with similar levels of deprivation.

## Notes

*This article meets the Accreditation Council for Graduate Medical Education and the American Board of Medical Specialties Maintenance of Certification competencies for Patient Care, Medical Knowledge, and Practice-Based Learning and Improvement*.
